# Competence of novice nurses: role of clinical work during studying

**Published:** 2015

**Authors:** H Manoochehri, E Imani, F Atashzadeh-Shoorideh, A Alavi-Majd

**Affiliations:** * Nursing Department, Shahid Beheshti University of Medical Sciences, Tehran, Iran; ** International Branch, Shahid Beheshti University of Medical Sciences, Tehran, Iran; *** Biostatistics Department, Shahid Beheshti University of Medical Sciences, Tehran, Iran

**Keywords:** nursing profession, student, clinical competence, work during studying, qualitative study

## Abstract

**Aim:** Clinical competence is to carry out the tasks with excellent results in a different of adjustments. According to various studies, one of the factors influencing clinical competence is work experience. This experience affects the integrity of students' learning experience and their practical skills. Many nursing students practice clinical work during their full-time studying. The aim of this qualitative research was to clarify the role of clinical work during studying in novice nurses' clinical competence.

**Methods:** This qualitative content analysis performed with the conventional approach. All teaching hospitals of Hormozgan University of Medical Sciences selected as the research environment. To collect data, deep and semi-structured interviews, presence in the scene and manuscripts used. To provide feedback for the next release and the capacity of the data, interviews were transcribed verbatim immediately.

**Results:** 45 newly-graduated nurses and head nurses between 23 and 40 with 1 to 18 years of experience participated in the study. After coding all interviews, 1250 original codes were derived. The themes extracted included: task rearing, personality rearing, knowledge rearing, and profession rearing roles of clinical work during studying.

**Conclusion:** Working during studying can affect performance, personality, knowledge, and professional perspectives of novice nurses. Given the differences that may exist in clinical competencies of novice nurses with and without clinical work experience, it is important to pay more attention to this issue and emphasize on their learning in this period.

## Introduction

Clinical competence described as functional efficiency and the ability to combine skills, knowledge, attitudes, and amounts in particular status of function [**1**]. Benner (1982) established competence of nurses as the capacity to work with desired results under different conditions of real work. According to Benner (1984), competence is a progressive experience over five stages: beginner, advanced beginner, competent, proficient and specialist. Nurses, who reach the stage of power, are consciously able to plan their actions but lack the flexibility and speed [**2**]. Benner (1984) identifies a competent nurse as being “on the job or in the identical status for 2 or 3 years” [**3**].

Nursing training plans combine skills for pupils to acquire competencies to satisfy the requirements of clinical training and enhance the flexibility of amateur therapists while they begin clinical conditions. These will intensify their morale, lowering turnover scores, and store the terms and cost for the re-education of novice nurses [**4**]. Evidence shows that nurses between 21 and 35 have the highest dissatisfaction from their work because they do not have the necessary experience, and they are not competent enough to meet the patients' needs and provide care with high quality [**5**]. 

Having competencies essential for nurses to provide care for patients [**6**]. Competence of nurses has direct effects on the safety and health of patients, and lack of it can result in medical mistakes and severe results for the patients [**7**]. Thus, the clinical performance and competence of novice nurses have been a main professional and corporate problem for providers and buyers of nursing care [**8**]. Furthermore, it is needed that the nurses show the capability to supply quality care for patients and to collaborate with other nurses and colleagues [**4**]. Competence assessment of novice nurses has been considered necessary [**9**]. 

Studies showed that many factors influence on the clinical competence of nurses and improvement of its quality. Wangensteen et al. (2012) demonstrated that such independent variables such gender, educational level, work experience, and activities are predictors for nursing competence. Several studies have shown that one of the factors influencing clinical competence is work experience [**10**-**12**]. Evidence shows that many nursing students work in clinical settings during their full-time study [**13**]. Figures show that 50 to 60 percent of students in Asian countries work [**14**]. Factors such as the financial motivation, personal satisfaction, skill development, experience gain, employment chance after graduation and familiarity with new friends attract them to work [**15**-**18**]. Meanwhile, some studies have emphasized on financial issues while others have found personal satisfaction and experience gains the main factors in this regard [**19**]. Also, work experience effects on socialization, values, attitudes and behaviors of students [**13**]. 

Recent findings suggest that clinical work and patient care can influence the totality of learning and practical education in students [**13**]. Working in students has such benefits as increased confidence, self-esteem, skills and understanding of the problems in the real job [**13**,**20**-**22**]. However, some disadvantages such as restrictions in living as well as social activities, lack of freedom to enjoy educational life, high level of physical and mental stress, role conflict, learning distraction from faulty routines, absence from classes, delay in home works, lack of time for studying and adverse effects on academic performance as well as quality of life [**13**,**19**,**21**,**23**-**27**].

The study of Salamonson and Andrew (2006) showed that 78% of students in Australia worked during studying, and half of them worked more than 16 hours a week. Some evidence also suggests that this amount of work puts them at risk for poor academic performance [**16**,**23**,**28**]. The study of Rochford et al. (2009) showed that the amount of working hours per week in nursing students is an important factor for predicting their academic performance and undesirable results in scores. Some researchers also reported poorer mental health status related to work during studying and its amount [**27**]. 

As mentioned above, it seems that working during studying can be useful on clinical competencies of new nurses. Studies show that these nurses are not ready enough to deal with clinical challenges [**29**-**32**], and the curriculum cannot prepare them sufficiently for free performance [**33**]. The study of Oermann and Garvin (2002) showed that new nurses were maybe weak in practical skills and, as a result, they experience stress such as lack of confidence and competence, medical errors due to increased workload and responsibilities; new situations, environments, and procedures; and staff shortages. Hartigan et al. (2010) in a qualitative research specified 41 challenging acute nursing seasons for novice nurses. They knew competencies required to manage these challenges included: case assessment, clinical and technical experiments, interactions as well as links. 

Several studies have shown that one of the factors influencing clinical competence is work experience [**10**-**12**,**34**,**35**], but still, the phenomenon of working during studying has not been adequately explained. Since there is no sufficient literature on this topic, the question is, despite all adverse consequences of work during studying, whether this type of work can play a role in clinical competence of novice nurses. On the other hand, due to staff shortages and the need for nursing services at hospitals, one of the new policies of the Ministry of Health in Iran is to recruit nursing students. Searching literature did not yield any study regarding the role of clinical work during studying on the clinical competence of novice nurses or the perception of nurses in this regard. Therefore, an in-depth study of this phenomenon was needed to understand its nature. Accordingly, a qualitative design was applied to answer the following question: what is the role of clinical work during studying in the competence of novice nurses? The objective of this research is to describe the role of clinical work during studying in the ability of novice nurses.

## Methods

Content analysis with conventional approach was employed. Deep semi-structured interviews with 45 nurses and nursing managers performed. Content analysis deals with the objective and systematic description of the manifest content of communication and interpretations of latent content [**36**,**37**]. Ethical approval deduced from the review board of the University (moral code: it Sbmu. iasb. rec No: 1000/ 271 date: 21 Jul 2014) and permission for four teaching hospitals gained from International Branch of the University. Considerations about informed consent, confidentiality, anonymity, information providing and handling seen. Mentioned data regarding the research supplied for the members, and they agreed a written consent. They informed of their ability to cancel from the research. 

Clinical nurses with the experience of work during studying or work with students and nursing managers participated. They were males and females from a variety of clinical settings. Interviews performed at teaching hospitals affiliated with Hormozgan University of Medical Sciences. Data collected in 2014 and 2015. In total, 45 nurses and nursing managers took part in individual interviews. To receive data, deep and semi-structured interviews, presence in the field and manuscripts used. An interview schedule according to a technical structure, review of literature and aims of the study devised. It included issues on nurses' percentage of the role of work during studying in clinical competence of novice nurses and an opportunity for open comments regarding problems that affected on their skill. Interviews were continued to reach the deep and sufficient data. Their durations ranged from 40 to 90 minutes.

Conventional content analysis was conducted [**38**] with open coding, category forming and abstracting. Immediately after each interview, the researchers listened to the recordings several times, so that they became similar with the content to have feedback for the next interview and adequacy of data. The transcripts read repeatedly. Field notes then added to the transcripts and sentences and sections were investigated to recognize potential fundamental ideas in any discussion. Subject imports with familiar objects arranged collectively. Abstract labels were assigned to these concepts and gradually a coding structure made that included the extent of views defined via the members. A schedule of roles of work during studying in clinical competence of novice nurses formulated.

To confirm the validity and accuracy of the study, credibility, dependability, conformability and transferability of data were assessed. To improve safety and efficacy of conclusions, a copy of the transcripts were got to a skill qualitative study to confirm the procedure of information investigation. Moreover, researchers invited the participants to view and comment on the list. This strategy both verified the results and considerably improved the rates of efficacy and safety of the information.

## Results

45 nurses and nursing managers between 23 and 40 participated in the study. Years of their skill among 1-18 years with an average of 5.7. Findings indicated that 22 of the participants had worked during studying. After coding all the interviews, 1250 original codes were developed. By the content investigation, four main elements emerged including six categories including task rearing, personality rearing, knowledge rearing and profession rearing roles of clinical work during studying in clinical competence of novice nurses (**Table 1**).

**Table 1:** Role of clinical work during studying on clinical competence of novice nurses from different dimensions

subcategories	categories	themes
Professional performance promotion	Functional role	task rearing
Improve caring performance		
Educational performance development		
Reduce incidence of medical errors	Outcome role	
Increase accuracy in work		
Increase efficiency		
Improve personality traits	Self refinement role	personality rearing
Increase sense of responsibility		
Develop critical thinking		
Increase efficacy		
Strength social relationships	relational role	
Increase management capabilities		
Increase scientific information	Knowledge development role	knowledge rearing
Enhance learning		
Increase awareness of professional issues	Professional role	profession rearing

**Task rearing role**

**Functional role**

Nurses believe that experience of work during studying can have some effects on performance of novice nurses. Since nurses' functions are very broad and cover all aspects of patient care, a clinical experience can also affect it. Therefore, work experience during studying has functional roles including improvement of professional practice, care provision as well as educational capabilities, and outcome roles including reduction of medical errors, increased accuracy, as well as efficiency.

The experience of work during studying can positively influence on professional practice, impact on professional actions of nurses, improve learning, and strengthen nursing activities. Nurses’ response to various issues during work will be affected as well.

"Working during studying makes students have responsibility in their job .... Makes them react faster. Because nurses’ reactions to issues can save patients' lives or fall them at risk, the results of students increase primarily at work during studying." (Participant number 31, female, 11 years of experience, 35 years old)

In the viewpoints of nurses, clinical work experience is effective in improving care provision both during studying and after graduation. This role also has many effects on improving the condition of patients and can even prevent serious problems in the care process.

"A nurse with more experience has more attention. Nurses should carefully observe everything in patients. Perhaps, if these changes are detected on time and reported to a doctor, many complications can be prevented or at least, the condition of patients would not get worse again." (Participant number 6, female, four years of experience, 27 years old)

One of the tasks of nurses is instruction. In a hospital, nurses instruct both patients and their novice colleagues and familiarize them with the rules of the ward. In contrast, since newly employed nurses usually have more updated scientific information, they may be able to instruct their colleagues. Work experience during studying is also useful in this role of nurses.

"When the issue is communication with patients, those with experience can better communicate. When preparing the patients for the operating room, training them during discharge, and positioning them, nurses with previous work experience are much more skillful because these tasks are familiar with them. So, they do these works better and spend less time." (Participant number 9, male, one year of experience, 23 years old)

**Outcome role**

By practicing nursing activities in the real workplace, students would have fewer errors in the future as novice nurses. Because students learn how to perform tasks, they work with less medical errors.

"Certainly, the nurses who have had work experience act more professional, have fewer mistakes and are more careful. Their work quality is good because when they work on the ward, their errors will be corrected and they gain more experience." (Participant number 11, male, one year of experience, 23 years old)

With different experiences, expert nurses can supervise their practice and students face with various learning opportunities to perform their tasks efficiently with accuracy and improve the quality of their work.

"If nurses have work experience, they would feel less stress when they begin to work .... Perform better work, spend more time talking with the patients, instruct them better, experience more collaboration, feel more interest in the job, and, on the whole, gain a greater return." (Participant number 22, female, head nurse, seven years of experience, 29 years old)

**Personality rearing role**

**Self-refinement role**

In the viewpoint of nurses, having clinical work experience during studying can develop one's personality and has self-refinement and relational roles. Having work experience makes head nurses, and other colleagues respect to novice nurses, and this, shape their character. Also, many characteristics are affected. They learn techniques of problems and face with them better.

"The work impacts on students’ personality and self-confidence. Having real experience makes them do the work more comfortably with more confidence. So, they do not feel shame in front of their colleagues because of incompleteness or frequent mistakes. They would find stronger characters." (Participant number 27, female, one year of experience, 23 years old)

Working during studying gives a sense of responsibility for patients to students. Strengthening this sense, in turn, leads to more individual consciousness and they try to provide better care for their patients. By observing correct and incorrect methods in patient care and working with different nurses, they gain great insight from these experiences, and their critical thinking skills would develop, and they can have better judgments to critique the functions of themselves and others. Thus, they can recognize and correct mistakes of their tasks and try to increase their capabilities, personality, performance, and their knowledge.

"It makes them work to empower themselves. Always, they study and have updated information, and even try to get a higher level education." (Participant number 14, female, three years of experience, 26 years old)

**Relational role**

Nursing students being among expert personnel and working with them makes socialization easier. As a result, when working after graduation, they can better adapt to their work environment and handle various situations.

"This experience makes students be familiar with the workplace. They know how to behave in different conditions or how to meet the needs of patients or staff. They observe the cooperation and coordination of nurses in shortage situation to cover all the shifts." (Participant number 22, female, head nurse, seven years of experience, 29 years old)

Working with a head nurse is an opportunity for students to learn about ward management. Their judgment and critical thinking skills are improved and, after graduation, they can manage their functions and experience less stress during the shifts in this regard. By working with expert personnel, their learning quality rises and many procedures and interventions are practiced. Thus, they understand how to set priorities during care.

"Nurses with experience have better managerial skills and updated knowledge. They manage patients better so that they can do all necessary works. They try to divide their time for different tasks and prioritize them." (Participant number 7, female, two years of experience, 25 years old)

**Knowledge rearing role**

Clinical experience can strengthen and internalize theoretical learning in the classroom. It called “knowledge rearing part” to reflect the viewpoints of nurses. It denotes that clinical experience increases the knowledge of students and updates their information. 

"Work during studying helps them learn. Many conditions and interventions they study in the class with no real imagination become sensible and tangible for them. It creates a better learning for them." (Participant number 18, female, four years of experience, 28 years old)

**Profession rearing role**

Work experience during studying helps students improve their professional identity. During the work, students can clearly observe many vocational and legal issues nurses deal with them. They may have some critiques about nurses’ reactions to such matters and learn correct methods of confrontation. When the students realize the consequences of these issues for their colleagues, they can acquire valuable experiences in this regard.

"When students closely work with nurses, they become familiar with the legal responsibilities of a nurse, and they may practically understand the problems created for them." (Participant number 24, female, two years of experience, 25 years old)

Given nurses with work experience during studying, this can improve the viewpoints of a student to the profession. Especially, those who have come to the nursing profession without previous knowledge will have better viewpoints to it. Nurses may even have the better incentive to make a better future. Some seek to promote their professional situation, and some tend to study for higher education as a goal and try to reach it.

"Work during studying can help person's permanent occupation. I mean nursing. It increases their experience and could encourage them to learn more. It makes them successful at present or in the future." (Participant number 31, female, 11 years of experience, 35 years old)

## Discussion 

The purpose of this research is to determine a role of clinical work during studying in the competence of novice nurses. The results showed different roles related to this type of work including functional, outcome, self-refinement, social, affective, knowledge development, and professional incompetence. These roles categorized in 4 domains including task rearing, personality rearing, experience rearing and profession rearing. 

Aari et al. (2008) determined 4 important areas of experimental competence including specific skill and knowledge, value, objective and test base of severe and essential treatment [**39**]. ParsaYekta et al. (2005) found that major dimensions of clinical competence in nursing profession include ethical and professional performance, creative thinking, assessment, and analysis of information, management of dependence and independence in nursing care, safety, and comfort of clients, effective communication, caring measures, education, profession progression as well as development, and cooperation with other health care staff [**35**]. 

In the study of Mohtashami et al. (2013) on assessment of clinical competence of psychiatric nursing students, four main themes emerged: preparation and familiarization, confrontation, involvement, and ability [**40**,**41**]. 

Nurses in this study believed that having clinical work experience could improve the performance of novice nurses. Philips et al. (2012) showed that advanced stage of ward skill and university experimental training in nurses allows the decision making advancement, skills proficiency and teamwork skill in the “real-world” configurations [**16**]. These findings also support the studies of Olsen (2001) and Nelson (2004) who proposed that undergraduate pupil worked in hospitals are better managed for a transient to real hospitality configurations and RN practice regarding the organization, confidence, and knowledge as well as skill acquisition. These studies indicated that advanced hospital skill aimed nurses in their view and socialization to that specified organizations [**42**,**43**]. Studies discussed that this procedure of socialization makes novice nurses need shorter time for orientation and ready to work [**44**-**45**].

Hasson et al. (2013) argue that experience of clinical work during studying may help in the various nurse. It also influences on values, attitudes, and behaviors [**13**]. Philips et al. (2012) believe that nurses with work experience during studying gain skills in time administration, dealing with hard person, self believe advancement, and association [**16**]. Curtis and Williams (2002) referring to these skills argued that they are transferrable to real clinical practice and can observe in future employment [**46**].

Hartigan et al. (2010) identified 41 main treatment episodes in experimental action and said that 4 considerations of competence are needed to control them: patient assessment, technical as well as clinical skills, interactions as well as communication, and clinical decision-making [**47**].

Our findings showed that work experience during studying can make newly graduated nurses ready for confronting these challenges, and they can acquire competencies for improving their performance. Wangesteen et al. (2012) also identified that work experience in novice nurses was a significant predictor for the competence of nurses [**12**]. However, the present study showed that work during studying can make them ready for nursing practice in real clinical settings and task rearing, personality rearing, knowledge rearing as well as profession rearing are evident roles for safe provision of care.

## Conclusion

Clinical experiences of nurses participating in this qualitative study identified four key aspects of competence. The results are not entirely novel, however they used to power the conclusions of international researches. They can assist to understand the role of clinical work experience during college life in clinical competence and what expected from novice nurses entering practice. Hence, it is vital to determine all views of this phenomenon. It seems that clinical work during studying can positively influence on the competence of currently graduated ones.

**Limitation**

The study took place in Province in Iran; therefore, the findings cannot be generalized. All the graduated nurses who take part in this research worked as registered ones in teaching hospitals. 

**Recommendation**

The aspect of nurses whom were not employed below graduation or worked in other hospitals not considered as it was out of the scope of this research. Their points of view may be various and taking this skill and information would be suitable for more research. Thus, using their viewpoints about work during studying is recommended.

**Acknowledgments**

We would like to thank the members, head nurses and everybody else who took part in the study and spent their time for the interviews and validation of findings. The paper is taken from a Ph.D. dissertation in nursing in the International Branch of Shahid Beheshti University of Medical Sciences, Tehran, Iran.

**Financial Disclosure**

The authors have no financial interests related to the material in the paper. 

**Funding/ Financial Support**

This study received no funding from any organization. 
